# 自体造血干细胞移植和单纯化疗治疗第一次完全缓解期结性外周T细胞淋巴瘤的队列分析

**DOI:** 10.3760/cma.j.issn.0253-2727.2021.05.014

**Published:** 2021-05

**Authors:** 阳 焦, 薇 刘, 树华 易, 慧敏 刘, 颖 于, 文阳 黄, 录贵 邱, 德慧 邹

**Affiliations:** 中国医学科学院、北京协和医学院血液病医院（中国医学科学院血液学研究所），实验血液学国家重点实验室，国家血液系统疾病临床医学研究中心，天津 300020 State Key Laboratory of Experimental Hematology, National Clinical Research Center for Blood Diseases, Institute of Hematology & Blood Diseases Hospital, Chinese Academy of Medical Sciences & Peking Union Medical College, Tianjin 300020, China

结性外周T细胞淋巴瘤（PTCL）是一类相对少见的异质性较强的非霍奇金淋巴瘤（NHL），常见亚型包括外周T细胞淋巴瘤非特指型（PTCL-NOS）、血管免疫母细胞性T细胞淋巴瘤（AITL）、ALK阳性间变大细胞淋巴瘤（ALK^+^ALCL）、ALK阴性间变大细胞淋巴瘤（ALK^−^ ALCL）。这些亚型侵袭性强，除ALK^+^ALCL外，其余亚型预后较差。常用的一线化疗方案为CHOP或CHOP样方案，治疗总体反应率（ORR）为70％～80％，但长期生存率仅30％～40％[1]–[3]。美国国立癌症网络（NCCN）指南推荐PTCL患者一线化疗完全缓解（CR_1_）后，采用大剂量化疗（HDT）联合自体造血干细胞移植（auto-HSCT）作为巩固治疗。由于缺乏随机对照研究，与单纯化疗相比，CR_1_患者巩固性auto-HSCT的价值和意义尚不明确[4]，国内缺少真实世界的相关研究数据。本研究采用1∶1匹配的队列分析比较一线auto-HSCT和单纯化疗对CR_1_期结性PTCL患者生存的影响。

## 病例与方法

1. 病例：研究纳入1992年6月至2019年12月就诊于中国医学科学院血液病医院的PTCL病例，纳入标准：①结性PTCL（除外ALK^+^ALCL）；②年龄15～65岁；③一线诱导化疗后获得完全缓解（CR）；④未接受一线异基因造血干细胞移植（allo-HSCT）巩固治疗。根据巩固治疗方式分为auto-HSCT组和化疗组。诊断分型采用2008年版WHO造血与淋巴系统肿瘤分类标准，临床分期采用Ann Arbor分期标准，体能状态评分采用美国东部肿瘤协作组（ECOG）评分系统。预后风险分层采用国际预后指数（IPI）和T细胞淋巴瘤预后指数（PIT），疗效评价采用2014年版Lugano非霍奇金淋巴瘤疗效评价标准。

2. 自体外周血干细胞动员、采集和移植方案：auto-HSCT组患者采用诱导化疗方案或依托泊苷联合G-CSF动员造血干细胞。预处理方案为BEAC（卡莫司汀+阿糖胞苷+依托泊苷+环磷酰胺）/BEAM（卡莫司汀+阿糖胞苷+依托泊苷+美法仑）或GBC（大剂量吉西他滨+白消安+环磷酰胺）方案。回输的中位单个核细胞（MNC）数为6.26（5.39～15.60）×10^8^/kg，回输的中位CD34^+^细胞数为4.26（2.15～21.33）×10^6^/kg。

3. 随访：随访截止日期为2019年12月31日。无进展生存（PFS）期定义为自疾病确诊至疾病进展、末次随访或各种原因死亡的时间。总生存（OS）期定义为自确诊至因各种原因死亡或末次随访的时间。

4. 统计学处理：采用SPSS 25.0和GraphPad Prism 8.0软件进行统计学分析与作图。采用*χ*^2^检验比较组间率和构成比，采用Kaplan-Meier法进行生存分析，采用Log-rank检验比较生存率。*P*<0.05为差异有统计学意义。

## 结果

1. 患者的基本临床特征：115例初诊结性PTCL患者完成诱导化疗并评估疗效，101例治疗反应达到≥部分缓解（PR），ORR为87.8％。其中49例（42.6％）患者获得CR，排除5例年龄65岁以上不适合移植的患者和2例接受allo-HSCT的患者，本研究共纳入42例结性PTCL患者，包括PTCL-NOS 34例（81.0％）、AITL 6例（14.3％）、ALK^−^ ALCL 2例（4.7％）。auto-HSCT组和化疗组各21例患者。auto-HSCT组患者中位年龄38（17～63）岁，化疗组中位年龄47（16～64）岁，两组的差异无统计学意义（*P*＝0.136）。两组患者在性别、年龄、病理亚型、有无B症状、ECOG评分、临床分期、LDH水平、结外病变数目、有无骨髓侵犯、IPI评分和PIT评分方面的差异均无统计学意义（表1）。

**表1:** 42例一线化疗完全缓解的结性外周T细胞淋巴瘤患者基本临床资料［例（％）］

	auto-HSCT组	化疗组	*χ*^2^值	*P*值
性别			0.467	0.495
男	14（66.7）	16（76.2）		
女	7（33.3）	5（23.8）		
年龄			0.778	0.663
<60岁	17（81.0）	19（90.5）		
≥60岁	4（19.0）	2（9.5）		
病理亚型			0	1.000
PTCL-NOS	17（81.0）	17（81.0）		
AITL	3（14.3）	3（14.3）		
ALK^−^ ALCL	1（4.7）	1（4.7）		
B症状			0	1.000
有	12（57.1）	12（57.1）		
无	9（42.9）	9（42.9）		
ECOG评分			0.099	0.753
≤1	12（57.1）	13（61.9）		
>1	9（42.9）	8（38.1）		
AnnArbor分期			0.227	1.000
Ⅰ～Ⅱ	2（9.5）	3（14.3）		
Ⅲ～Ⅳ	19（90.5）	18（85.7）		
LDH			0.889	0.346
正常	11（52.4）	14（66.7）		
升高	10（47.6）	7（33.3）		
结外病变数目			2.100	0.488
≤1	19（90.5）	21（100.0）		
>1	2（9.5）	0（0）		
骨髓侵犯			0.096	0.757
有	11（52.4）	12（57.1）		
无	10（47.6）	9（42.9）		
IPI评分			3.482	0.357
0～1	5（23.8）	9（42.8）		
2	7（33.3）	8（38.1）		
3	8（38.1）	3（14.3）		
4～5	1（4.8）	1（4.8）		
PIT评分			2.059	0.587
0	3（14.3）	3（14.3）		
1	8（38.1）	6（28.6）		
2	9（42.8）	8（38.1）		
3～4	1（4.8）	4（19.0）		

注：auto-HSCT：自体造血干细胞移植；PTCL-NOS：外周T细胞淋巴瘤非特指型；AITL：血管免疫母细胞性T细胞淋巴瘤；ALKALCL：ALK阴性间变大细胞淋巴瘤；ECOG：美国东部肿瘤协作组；IPI：国际预后指数；PIT：T细胞淋巴瘤预后指数

2. 一线化疗方案：17例（40.5％）患者采用CHOP（环磷酰胺+长春新碱+阿霉素+泼尼松）方案，8例（19.0％）患者采用ED（P）OCH（依托泊苷+长春新碱+阿霉素+环磷酰胺+地塞米松或泼尼松）方案，6例（14.3％）患者采用ED（P）OCH、GDPE（吉西他滨+地塞米松+顺铂+依托泊苷）交替方案，其余11例（26.2％）患者采用其他方案，主要包括HyperCVAD（环磷酰胺+长春新碱+阿霉素+地塞米松）/MA（甲氨蝶呤+阿糖胞苷）、VDCLP（长春新碱+柔红霉素+环磷酰胺+泼尼松+左旋门冬酰胺酶）、GDPE等。auto-HSCT组和化疗组在一线化疗方案种类上的差异无统计学意义（*P*＝0.814）。auto-HSCT组一线化疗中位疗程数5（4～9）个，化疗组中位疗程数6（4～13）个，差异无统计学意义（*P*＝0.398）。

3. 整组患者的生存分析：中位随访50（4～151）个月，其中17例（40.5％）患者疾病复发并均已死亡；3例（7.1％）失访；其余22例（52.4％）仍存活。全组患者的中位PFS期为48（2～151）个月，4年PFS率为（47.9±10.0）％，中位OS期为60（4～151）个月，4年OS率为（56.0±9.3）％。

4. auto-HSCT组和化疗组生存分析比较：auto-HSCT组中位随访53（8～151）个月，6例（28.6％）患者移植后疾病复发，移植后中位复发时间为12.5（3.0～34.0）个月；7例（33.3％）患者死亡，死亡原因分别为疾病进展（5例）、感染（1例）和急性重症肝炎（1例），其余14例仍存活。auto-HSCT组中位PFS期和OS期均未达到，4年PFS率和OS率分别为（59.8±13.3）％和（67.7±12.3）％。化疗组中位随访37（7～139）个月，11例（52.4％）患者疾病复发，其中3例（14.3％）失访，余8例（38.1％）存活；10例（47.6％）患者死亡，其中9例死于疾病进展，另1例死于严重感染。化疗组中位PFS期和OS期分别为23.3（2.0～130.0）个月和36.0（7.0～139.0）个月，4年PFS率和OS率分别为（32.4±15.5）％和（44.4±12.9）％。auto-HSCT组和化疗组PFS期和OS期的差异均有统计学意义（图1）。

**图1:**
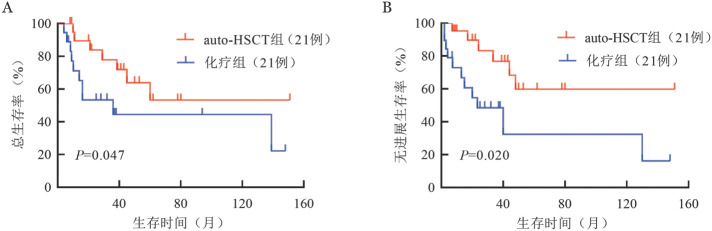
自体造血干细胞移植（auto-HSCT）组和化疗组结性外周T细胞淋巴瘤患者的总生存（A）和无进展生存（B）曲线

5. 预后因素分析：单因素预后分析显示，ECOG评分>1、PIT评分>1和未行一线巩固性auto-HSCT是患者PFS和OS不良的预后因素，合并骨髓侵犯和IPI评分≥2（特别是≥4）是OS不良的预后因素。性别、年龄、病理亚型、有无B症状、疾病分期、LDH水平和一线化疗方案种类均未显示预后意义（表2）。

**表2:** 一线化疗完全缓解的结性外周T细胞淋巴瘤患者总生存（OS）和无进展生存（PFS）的单因素分析（％，*x*±*s*）

因素	例数	OS			PFS		
		4年OS率	*χ*^2^值	*P*值	4年PFS率	*χ*^2^值	*P*值
ECOG评分			19.133	<0.001		12.461	<0.001
≤1	25	90.9±6.1			71.1±11.8		
>1	17	8.5±8.0			9.3±8.8		
骨髓侵犯			4.261	0.039		1.691	0.193
是	23	44.5±11.5			35.6±12.6		
否	19	71.8±15.1			63.8±14.5		
IPI评分			6.526	0.038		5.708	0.058
0～1	14	90.9±8.7			67.5±17.2		
2～3	26	45.8±11.8			42.2±12.4		
4～5	2	0			0		
PIT评分			9.242	0.002		8.700	0.003
≤1	20	93.8±6.1			77.7±12.3		
>1	22	26.3±11.5			20.7±12.5		
一线巩固性auto-HSCT			3.942	0.047		5.406	0.020
是	21	67.7±12.3			59.8±13.3		
否	21	44.4±12.9			32.4±15.5		

注：ECOG：美国东部肿瘤协作组；IPI：国际预后指数；PIT：T细胞淋巴瘤预后指数；auto-HSCT：自体造血干细胞移植

23例合并骨髓侵犯患者中，12例接受auto-HSCT巩固治疗，另11例患者接受单纯化疗。auto-HSCT组和化疗组的4年PFS率分别为（54.0±17.3）％和0（*P*＝0.012），差异具有统计学意义；两组患者4年OS率分别为（61.4±15.3）％和（26.7±15.0）％（*P*＝0.055）。

## 讨论

本研究是国内首个真实世界、匹配的队列研究，比较了一线auto-HSCT和单纯化疗对CR_1_期结性PTCL患者生存的影响，并进行了预后因素分析。结果显示，一线auto-HSCT巩固治疗显著改善CR_1_期结性PTCL患者的PFS和OS。

结性PTCL往往是侵袭性淋巴瘤，除ALK^+^ALCL以外目前缺乏标准的治疗策略，CHOP或CHOP样方案仍然是目前一线治疗最常采用的诱导方案，但总体疗效欠佳[1]–[3]。多项回顾性和前瞻性研究显示一线auto-HSCT提高疗效[5]–[7]，因而推荐4～6个疗程诱导化疗后获得≥PR的适合移植患者序贯auto-HSCT巩固治疗[8]。近年研究结果进一步显示一线auto-HSCT巩固治疗的主要获益人群为获得CR_1_的患者。美国纪念斯隆凯瑟琳（Memorial-Sloan Kettering）癌症中心的一项研究纳入了112例结性PTCL患者[9]，均接受CHOP/CHOP样方案诱导化疗和auto-HSCT巩固治疗，结果显示中期PET-CT阴性患者有更好的生存，多维尔（deauville）5分法评分（i5PS）≤3分的患者较4～5分患者有更长的中位无事件生存（EFS）期（104个月对19个月，*P*<0.001）和中位OS期（64个月对11个月，*P*<0.001），支持CR_1_期结性PTCL患者接受auto-HSCT生存获益。因此NCCN、美国细胞治疗和移植学会（ASTCT）的指南目前均推荐于CR_1_期接受auto-HSCT巩固治疗[10]。

既往研究显示auto-HSCT前获得CR是与疗效最为相关的因素，然而诱导化疗后获得CR提示患者对化疗的敏感性更高，同时有更大概率接受后续auto-HSCT巩固治疗，故该结论可能存在选择性偏倚。LYSA研究组回顾性分析了来自法国、比利时和葡萄牙的269例年龄≤65岁，诱导治疗后获得≥PR的患者，根据意向治疗，145例和135例患者分别接受了auto-HSCT巩固和单纯化疗，然而无论是Cox回归多因素分析还是倾向评分分析，均未显示诱导治疗获得≥PR的患者从序贯auto-HSCT治疗中获益[4]。另一项美国多中心COMPLETE研究纳入了119例CR_1_期结性PTCL患者，其中36例接受了auto-HSCT巩固治疗[11]。中位随访2.8年，auto-HSCT组和未移植组的OS时间分别为未达到和57.6个月（*P*＝0.06）。多因素分析显示，auto-HSCT是改善患者OS时间的独立影响因素，特别是AITL亚型、疾病晚期和IPI评分中高危及高危患者，接受auto-HSCT的患者较未接受auto-HSCT的患者具有更好的PFS和OS。本研究的结果也支持auto-HSCT巩固治疗能够改善CR_1_期患者的生存。

我们的结果再次验证了既往的研究结果，显示合并骨髓侵犯是影响生存的不良预后因素[12]，而auto-HSCT巩固治疗可显著改善骨髓侵犯患者的生存。本研究同时显示PIT评分较IPI评分能更好地预测PFS和OS。

尽管存在多种局限性，如回顾性分析、样本量偏小及非随机临床研究等，我们的队列研究结果支持结性PTCL（除外ALK^+^ALCL）患者诱导治疗获得CR后序贯auto-HSCT巩固治疗进一步改善了患者的生存。未来需要更多、更大系列的前瞻性研究，结合更精准的治疗反应评价方法和组织、分子标志等，以评价不同亚型特别是新药时代结性PTCL患者一线行auto-HSCT的价值和意义。
